# Increased Mast Cell Counts and Degranulation in Microscopic Colitis

**DOI:** 10.1155/2020/9089027

**Published:** 2020-01-04

**Authors:** Zhikai Chi, Jing Xu, Romil Saxena

**Affiliations:** ^1^Department of Pathology, University of Texas Southwestern Medical Center, Dallas, Texas, USA; ^2^Department of Pathology and Laboratory Medicine, Indiana University School of Medicine, Indianapolis, Indiana, USA

## Abstract

**Objectives:**

Microscopic colitis (MC) is characterized by chronic diarrhea, normal colonoscopy findings, and mucosal inflammation in colonic biopsies and can be classified as collagenous colitis (CC) or lymphocytic colitis (LC). However, the pathogenesis of MC is largely unknown. In this study, we aimed to study mast cell counts and activation in MC.

**Methods:**

We investigated 64 biopsy samples from the surgical pathology database of Indiana University Health, which met the diagnostic criteria for CC or LC along with 20 control samples collected from 2014 to 2015. The specimens were used for the quantification of mast cells by examining the presence of intracellular and extracellular tryptase by immunohistochemistry.

**Results:**

In the lamina propria, the mast cell count was higher in both CC and LC groups than the control (mean highest count, 39/high-power field (HPF) vs. 30/HPF vs. 23/HPF; *P* < 0.01). Extracellular tryptase was present in 10% of control subjects as compared to 41% of CC (*P* < 0.01). Extracellular tryptase was present in 10% of control subjects as compared to 41% of CC (*P* < 0.01). Extracellular tryptase was present in 10% of control subjects as compared to 41% of CC (

**Conclusions:**

The increased mast cell count and degranulation are identified in MC, suggesting that mast cell activation might be involved in the pathogenesis of MC.

## 1. Introduction

Microscopic colitis (MC) is an umbrella term for a disorder characterized by chronic diarrhea, normal colonoscopy appearance, and mucosal inflammation in colonic biopsies [1]. The pathogenesis of MC is not well understood and likely to be multifactorial. It was well established that MC was associated with nonsteroidal anti-inflammatory drug (NSAID) use, proton pump inhibitor (PPI) use, and smoking; in addition, there were recent evidences of the association with selective serotonin reuptake inhibitor (SSRI), statin, certain types of HLA, and various autoimmune diseases [2–7]. The two best-defined subtypes of MC are collagenous colitis (CC) and lymphocytic colitis (LC). The latter is histologically defined by intraepithelial lymphocytosis, mucosal surface damage and mucin loss, and expanded lamina propria (LP) with lymphoplasmacytic infiltration [8, 9]. The key histological feature of CC is the deposition of an abnormally thickened collagen layer underneath the surface epithelium [10, 11]. Clinical presentation of both subtypes includes chronic watery diarrhea, abdominal pain, and weight loss [12]. Corticosteroids (budesonide) are considered as the first-line treatment for patients with severe symptoms [13, 14]. However, the relapse rate after discontinuation of budesonide is high, and development of corticosteroid dependency and adverse side effects has been observed with long-term treatment [15].

Mast cells had been implicated in a variety of gastrointestinal disorders including mastocytic enterocolitis, allergic mastocytic gastroenteritis and colitis, chronic diarrhea in rheumatoid arthritis, and chronic diarrhea of unknown etiology [16–20]. In the largest study to date on colonic mast cells, mast cell counts were found to be elevated in patients with chronic diarrhea of unknown etiology, although the mast cell count had little diagnostic utility for that particular disease [18]. In this study, we investigated whether mast cells were involved in the pathogenesis of MC, which had not been previously examined. Tryptase was stored in secretory granules of mast cells and was released when mast cells degranulated [20], serving as a marker for this process [21]; therefore, we aimed to investigate the levels of this marker in MC. We hoped to provide evidences that would justify the measurement of these parameters to monitor treatment efficacy if anti-mast cell therapy was to be implemented in the future.

## 2. Methods

### 2.1. Patients

The study was approved by the Institutional Review Board of Indiana University. A total of 64 biopsy cases in the surgical pathology database of Indiana University Health from 2014 to 2015 that met the diagnostic criteria of CC or LC were analyzed in this study, along with samples from 20 healthy control subjects from the same time period. All LC or CC patients carried clinical suspicions of microscopic colitis with chronic watery nonbloody diarrhea (usually >6 months) and often with clinical requests to rule out microscopic colitis. The surgical pathology database of all patients was reviewed to make sure that only first-time diagnosis cases of CC or LC were included, and patients with inflammatory bowel disease or infection were excluded. Relevant clinical history was also reviewed, when available, for known risk factors of MC including medications, smoking history, and other autoimmune diseases. The control group is comprised of only healthy individuals who were referred for colorectal cancer screening and whose biopsies were diagnosed as colonic mucosa with no significant pathological changes.

### 2.2. Histomorphological Analysis

All biopsies were submitted to surgical pathology as “random biopsies” in one tissue jar containing multiple fragments from the cecum to the rectum. Routinely processed formalin-fixed, paraffin-embedded, hematoxylin and eosin- (H&E-) stained slides from MC patients and controls were reviewed by two pathologists (ZC and RS) to confirm the original diagnosis and evaluate inflammation. Histomorphological features were assessed under a light microscope (0.55 mm diameter, BX51; Olympus, Tokyo, Japan). In brief, LC showed increased intraepithelial lymphocytes, surface mucin loss, and expanded lamina propria by chronic inflammation that consists of lymphocytes and plasma cells, while CC additionally showed deposition of an abnormal layer of collagen underneath the surface epithelium which can be highlighted by Trichrome stain in difficult cases.

### 2.3. Immunohistochemical Analysis

Mast cell tryptase in formalin-fixed, paraffin-embedded tissue blocks was detected by immunohistochemistry as previously described [20, 21]. Briefly, deparaffinized tissue sections were labeled with a mouse monoclonal anti-mast cell tryptase antibody (1 : 1 dilution; Dako, Carpinteria, CA, USA). A high pH buffer solution in the PT module (Dako) was used for antigen retrieval, followed by incubation for 10 min each with primary antibody, Envision FLEX+M linker (Dako), Envision FLEX/horseradish peroxidase (Dako), and diaminobenzidine. In MC patient samples, only fragments with inflammation were evaluated. Mast cells in a single high-power field (HPF) with the highest or lowest cell density at a magnification of 400x were counted under a light microscope in a 0.55 mm area. Every effort was made to count sections of correctly oriented or close to correctly oriented colonic mucosae. Extracellular *β*-tryptase was assessed at a magnification of 400x under a light microscope and recorded as either “presence” or “absence.” Representative fields were selected and imaged at 100x and 400x magnification with the same microscope.

### 2.4. Statistics

Categorical data were analyzed with the *χ*^2^ test, and continuous data were analyzed with Student's *t*-test. *P* < 0.05 was considered statistically significant.

## 3. Results

### 3.1. Characteristics of the Study Population

The 20 control subjects had an average age of 61 years (range: 52–73 years) and included 14 females (70%); the 29 CC patients had an average age of 68 years (range: 49–89 years), with 26 females (90%); and the 35 LC patients had an average age of 69 years (range: 45–93 years), with 24 females (68%) (Table 1). There were no differences in the average age or sex ratio among the three groups (*P* > 0.05). Among 13 CC patients with relevant clinical history, 3 patients had known history of NSAID use, 3 with PPI use, and 2 with smoking history. While in 11 LC patients with relevant clinical history, 2 patients had known history of NSAID use, 2 with PPI use, and 2 with smoking history (Table 2). No known history of other MC risk factors was noted.

### 3.2. Mast Cell Counts Were Elevated in MC

**Figure 1:**
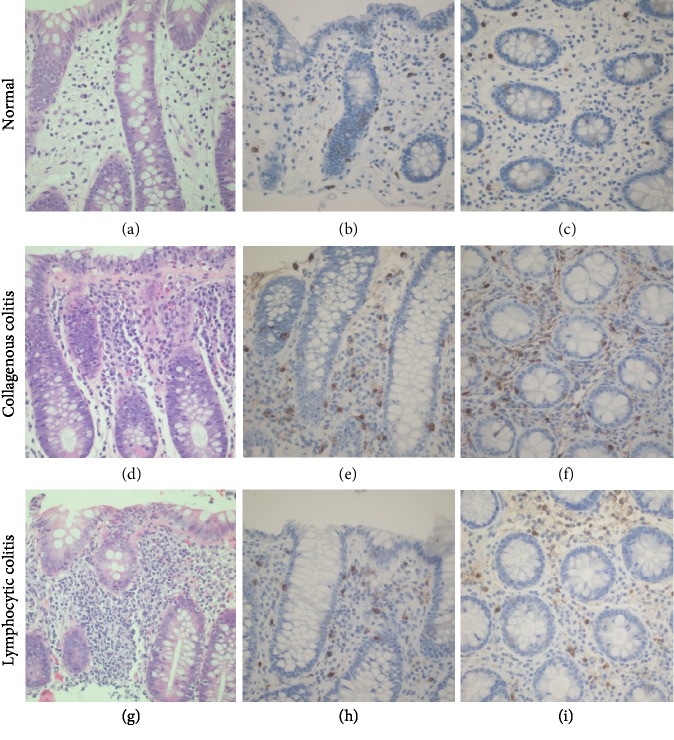
Representative images of colon biopsies and mast cell count areas: (a–c) control patients; (d–f) CC patients; (g–i) LC patients; (a, d, g) H&E staining; (b, c, e, f, h, i) immunohistochemical detection of *β*-tryptase; (b, e, h) highest mast cell counts in correctly orientated sections; (c, f, i) highest mast cell counts in tangential sections. Magnification = 400x (a–i).

Only colonic fragments with inflammation were included in mast cell quantification. Mast cells were counted in a single high-power field (HPF) with either the highest or the lowest cell density. Cells in the LP, muscularis mucosae, and submucosae were counted separately. The average highest mast cell counts in the LP were lower in controls (23/HPF, range: 10–37) than in the CC group (39/HPF, range: 19–63; *P* < 0.001) and the LC group (30/HPF, range: 13–42; *P* < 0.01) (Table 3 and Figure 1). The same trend was observed for the lowest mast cell counts in the LP (12/HPF, range: 3–25 vs. 20/HPF, range: 8–45 (*P* < 0.01) vs. 17/HPF, range: 6–27 (*P* < 0.01)). There were no differences in mast cell counts in the muscularis mucosae and submucosae among groups. Mast cell counts were correlated with NSAID use, PPI use, and smoking history; no difference was noticed with any of these risk factors (Table 4).

### 3.3. Extracellular Mast Cell Tryptase Is Increased in MC

**Figure 2:**
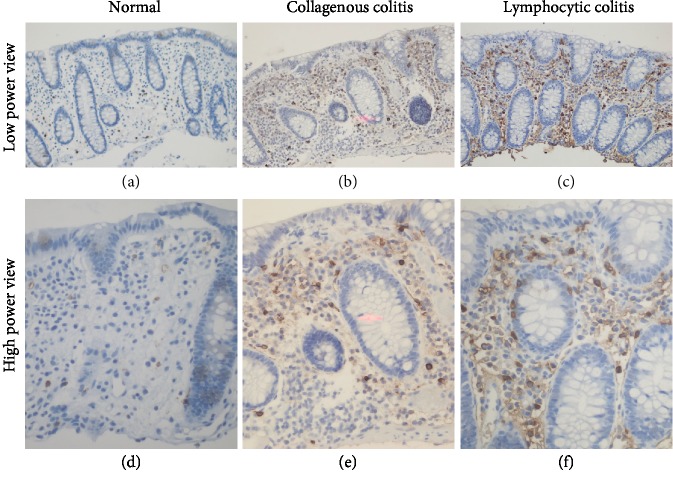
Immunohistochemical detection of extracellular mast cell tryptase in colon biopsies: (a, d) control patients; (b, e) CC patients; (c, f) LC patients. Magnification = 100x (a–c) and 400x (d–f).

We detected extracellular tryptase in 2 control patients (10%) as compared to 12 CC patients (41%; *P* < 0.05) and 21 LC patients (60%; *P* < 0.001) (Table 5 and Figure 2).

### 3.4. Mast Cell Counts Were Correlated with Extent/Scope of Inflammation in LC

**Figure 3:**
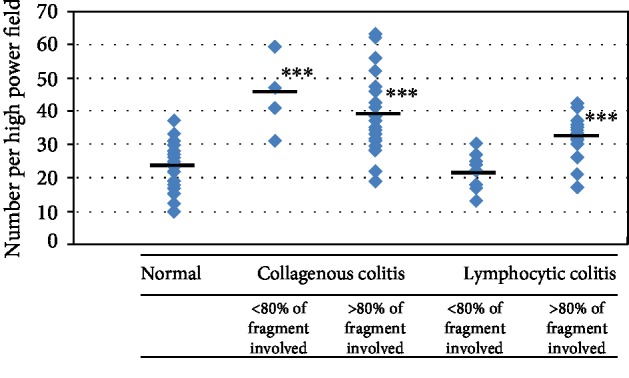
Highest mast cell counts in LP affected by the scope/extensiveness of inflammation. Magnification = 400x. HPF: high-power field. ^∗∗∗^*P* < 0.001 vs. control (Student's *t*-test). Control (*n* = 20); collagenous colitis (*n* = 29); lymphocytic colitis (*n* = 35).

MC is a patchy disease and may involve all or just part of the colon [11]. In the CC group, 93% (range: 63%–100%) of fragments were affected by inflammation whereas in the LC group, 89% (range: 44%–100%) were affected (Table 6). When the highest mast cell counts in LP were stratified into two groups, i.e., patients with <80% fragments involved by inflammation versus those with >80% fragments involved, a difference in mast cell counts was noticed in LC patients where the mast cell count in the <80% involvement group was 22/HPF (range: 13–30; *n* = 8; *P* > 0.05 vs. control) whereas in the >80% involvement group, the count was 32/HPF (range: 17–42; *n* = 27; *P* < 0.001 vs. control). However, no difference in the mast cell count was noticed in CC patients where the mast cell count in the <80% involvement group was 45/HPF (range: 31–59; *n* = 4) vs. 38/HPF (range: 19–63; *n* = 25) in the >80% involvement group, as both values were significantly higher than those in control subjects (*P* < 0.001; Table 4). The distributions of individual counts are shown in Figure 3.

## 4. Discussion

In this study, we report for the first time an elevation in the number of mast cells in MC patients. Mast cell counts have been investigated in association with a variety of GI disorders. Although no consensus regarding baseline mast cell counts and significant increase of mast cell counts had been established, our baseline mast cell counts in control subjects were mostly consistent with those reported in the most comprehensive of these studies [18, 20]. Although the quantitative analysis was performed using correctly oriented sections, mast cell numbers were elevated even in sections cut in the tangential plane. Furthermore, we observed that MC patients showed higher levels of diffuse extracellular tryptase, a marker of mast cell degranulation, than control subjects. Taken together, these results suggest that mast cell activation might be involved in the pathogenesis of MC.

There is no current consensus on how to grade the severity of MC's inflammation. Although active crypt inflammation and rare crypt abscess formation can be seen in MC, they were not the predominant or characteristic features of MC [22]. On the other hand, it is straightforward to describe the extent/scope of inflammation of MC. The threshold of 80% means that 8 out of 10 tissue fragments are involved by inflammation. It is a straightforward and reproducible way to describe inflammatory involvements. In LC patients, 80% was noticed because there was distinct difference in mast cell counts in patients with <80% inflammatory involvements compared with those with >80% inflammatory involvements. We hope that it will help pathologists make the decision whether to order tryptase immunostains in the clinical practice. Certainly, a larger-scale multicenter study will be needed to verify this threshold.

Both CC and LC are patchy diseases as they may involve all or just part of the colon [11]. It will be also interesting to know the correlation of mast cell counts with which portion of the colonic mucosa the biopsy was taken, as “optimum detection” of MC had been described to perform a full colonoscopy with two or more biopsies each from the right, transverse, descending, and sigmoid colon, in addition to sampling of endoscopically visible abnormalities [23]. However, all “random colon” biopsies in this retrospective study were submitted to surgical pathology in one tissue jar containing multiple fragments from the cecum to the rectum. Therefore, no analysis about localization can be performed. A future prospective study will be needed to address this interesting question.

Mast cells had been implicated in a variety of gastrointestinal disorders including mastocytic enterocolitis, allergic mastocytic gastroenteritis and colitis, chronic diarrhea in rheumatoid arthritis, and chronic diarrhea of unknown etiology [16–20]. Wide varieties of anti-mast cell therapy, including but not limited to H1-antihistamines and mast cell stabilizers [24], were FDA approved and can be readily used for future MC clinical trials. We showed that the increased number of mast cells in MC patients, and additionally, in LC patient mast cell counts, was correlated with the extent/scope of inflammation. These findings had practical implications; if anti-mast cell therapies were to be used to treat LC, patients with almost complete involvement of inflammation are presumably more suitable targets than those with less severe disease. In fact, our results showed a significant overlap in the mast cell counts of MC patients and control subjects. As such, mast cell quantification and analyses of extracellular mast cell tryptase levels and degree of inflammation are more useful for predicting individual responses to antimast cell therapies than for diagnosing MC. For the latter purpose, routine H&E staining along with clinical history and endoscopic findings should be more than adequate [11]. Despite the limits of our study, which include small biopsy specimen sizes, retrospective analysis, and no localization analysis, our results indicated that further investigations of the role of mast cells in microscopic colitis were warranted.

## Figures and Tables

**Table 1:** Demographics of the study population.

	Mean age (range)	Percentage of females (number)
Control (*n* = 20)	61 (52–73)	70% (14)
Collagenous colitis (*n* = 29)	68 (49–89)	90% (26)
Lymphocytic colitis (*n* = 35)	69 (45–93)	68% (24)

**Table 2:** Risk factors of microscopic colitis in the study population with available relevant clinical history.

	NSAID use	PPI use	Smoking history
Collagenous colitis (*n* = 13)	3 (23%)	3 (23%)	2 (15%)
Lymphocytic colitis (*n* = 11)	2 (18%)	2 (18%)	2 (18%)

Values are presented as number (percentage). NSAID: nonsteroid anti-inflammatory drug; PPI: proton pump inhibitor (PPI).

**Table 3:** Mast cell counts by tryptase immunohistochemistry.

	Highest count/HPF			Lowest count/HPF		
	LP	MM	SM	LP	MM	SM
Control (*n* = 20)	23 (10–37)	1 (0–1)	10 (3–22)	12 (3–25)	0 (0–1)	6 (3–11)
CC (*n* = 29)	39^∗∗∗^ (19–63)	1 (0–3)	15 (4–37)	20^∗∗^ (8–45)	0 (0–1)	8 (1–25)
LC (*n* = 35)	30^∗∗^ (13–42)	1 (0–3)	12 (3–26)	17^∗∗^ (6–27)	0 (0–1)	7 (0–21)

Values are presented as mean (range). ^∗∗^*P* < 0.01, ^∗∗∗^*P* < 0.001 vs. control (Student's *t*-test). HPF: high-power field; LP: lamina propria; MM: muscularis mucosae; SM: submucosae; CC: collagenous colitis; LC: lymphocytic colitis.

**Table 4:** Mast cell count correlation with well-established risk factors of microscopic colitis in the study population with available relevant clinical history.

	Mean highest count in lamina propria (range, number)	
	NSAID	No known NSAID
Collagenous colitis (*n* = 13)	42 (34-47, 3)^n.s.^	44 (29-63, 10)
Lymphocytic colitis (*n* = 11)	32 (21-42, 2)^n.s.^	30 (17-41, 9)
	PPI	No known PPI
Collagenous colitis (*n* = 13)	43 (31-63, 3)^n.s.^	43 (29-62, 10)
Lymphocytic colitis (*n* = 11)	30 (26-33, 2)^n.s.^	30 (17-42, 9)
	Smoking history	No known smoking history
Collagenous colitis (*n* = 13)	43 (34-52, 2)^n.s.^	43 (29-63, 11)
Lymphocytic colitis (*n* = 11)	33 (32-33, 2)^n.s.^	30 (17-42, 9)

n.s.: *P* > 0.05 (Student's *t*-test), patients with the risk factor versus patients without. NSAID: nonsteroid anti-inflammatory drug; PPI: proton pump inhibitor (PPI).

**Table 5:** Immunohistochemical detection of extracellular tryptase.

	Number (percentage)
Control (*n* = 20)	2 (10%)
Collagenous colitis (*n* = 29)	12 (41%)^∗^
Lymphocytic colitis (*n* = 35)	21 (60%)^∗∗∗^

^∗^ *P* < 0.05, ^∗∗∗^*P* < 0.001 vs. control (*χ*^2^ test).

**Table 6:** Mast cell counts in fragments involved by inflammation.

	Mean percentage of involved fragments (range)	Mean highest count in lamina propria (range, number)	
		<80% involvement	>80% involvement
CC (*n* = 29)	93% (63%–100%)	45^∗∗∗^ (31–59, 4)	38^∗∗∗^ (19–63, 25)
LC (*n* = 35)	89% (44%–100%)	22 (13–30, 8)	32^∗∗∗^ (17–42, 27)

^∗∗∗^ *P* < 0.001 vs. control (Student's *t*-test). CC: collagenous colitis; LC: lymphocytic colitis.

## Data Availability

The data used to support the findings of this study are available from the corresponding author upon request.
